# Effects of palmitic acid and high oleic acid soybeans on milk production of high-producing dairy cows

**DOI:** 10.3168/jdsc.2026-1130

**Published:** 2026-09-07

**Authors:** Alycia M. Bales, Viviane Ribeiro Pinheiro Coelho, Adam L. Lock

**Affiliations:** Department of Animal Science, Michigan State University, East Lansing, MI 48824

## Abstract

The objective of our study was to determine how the supply of dietary palmitic acid (C16:0) and high-oleic acid soybeans affected milk fat yield and milk fatty acid (FA) sources in high-producing dairy cows. Twenty multiparous Holstein cows (45.9 ± 4.7 kg/d of milk; 136 ± 18 DIM) were randomly assigned to treatment sequences in a replicated 4 × 4 Latin square design with 14-d periods. Treatments were (1) control with no additional C16:0 or high-oleic soybeans (CON), (2) 1.5% DM C16:0-enriched prill (PA), (3) 16% DM roasted and ground high-oleic soybeans (HOS), and (4) PA + HOS. To maintain dietary nutrients across all treatments, PA replaced soybean hulls and HOS replaced conventional soybean meal, bypass soybean meal, and soybean hulls for an average diet composition (% DM) of 21% forage NDF, 25% starch, and 17% CP. The total FA content of the treatments was 1.56%, 2.92%, 4.03%, and 5.42% DM, for CON, PA, HOS, and PA + HOS, respectively. The statistical model included the random effect of cow within square, the fixed effects of period, PA, and HOS, and the interaction of PA × HOS. No interactions were observed for DMI or milk production responses except for milk protein content. Diets containing PA increased the yields of 3.5% FCM, ECM, and milk fat, tended to increase milk yield, but did not affect DMI or milk protein yield. Diets containing HOS increased DMI and yields of milk, 3.5% FCM, ECM, milk fat and milk protein. For milk FA sources, there was no effect of PA or HOS for de novo milk FA yield. The PA-fed cows had increased mixed milk FA yield, whereas HOS-fed cows had decreased mixed milk FA yield. Diets containing PA did not affect preformed milk FA yield, whereas treatments with HOS increased yields of preformed milk FA. In summary, both PA and HOS increased yields of milk and milk fat. When combined, we observed an additive effect as demonstrated with milk fat yield, which increased by 90, 170, and 310 g/d for PA, HOS, and PA + HOS, respectively, compared with CON. These results demonstrate that combining complementary FA sources is an effective nutritional strategy to increase milk fat synthesis and yield in high-producing dairy cows.

## Graphical Abstract

**Figure:**
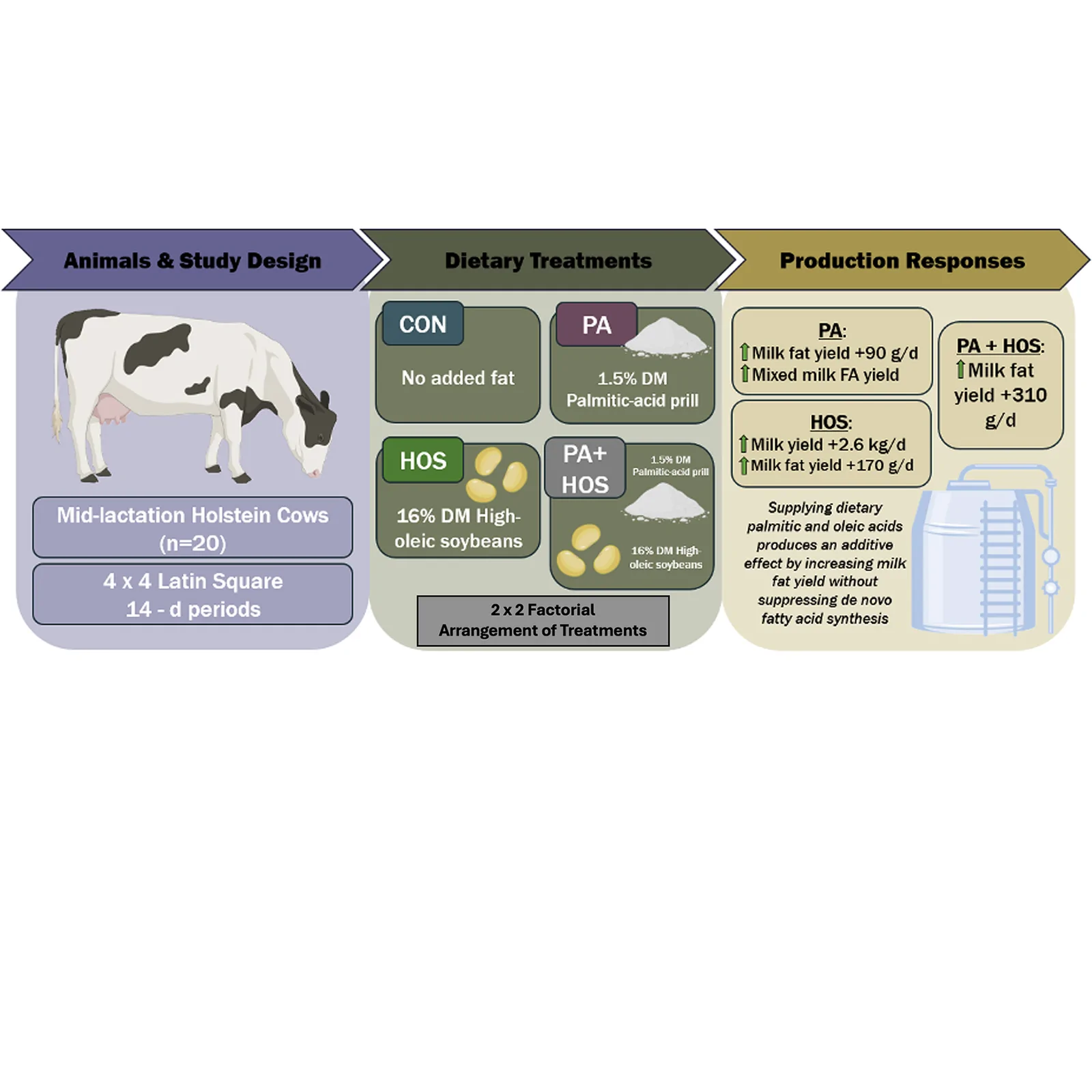

Dietary palmitic acid (C16:0) supplementation is consistently shown to improve milk fat yields across lactation stages, including fresh (de Souza and Lock, 2019; Parales-Girón et al., 2025) and mid-lactation cows (Rico et al., 2014; de Souza and Lock, 2018; de Souza et al., 2018). A recent meta-analysis reported that supplementing diets with C16:0 at an average inclusion rate of 1.8% of DM increased ECM by ∼2.0 kg/d and milk fat yield by ∼100 g/d compared with unsupplemented diets (dos Santos Neto et al., 2021). This benefit is partly explained by the role of C16:0 as the predominant fatty acid (**FA**) in milk fat and its contribution to mammary triglyceride (**TG**) assembly (Moore and Christie, 1979; Benoit et al., 2024). Indeed, feeding C16:0 has been shown to increase 16-carbon (mixed-source) milk FA and certain short- and long-chain FA (e.g., C4:0, *cis*-9 C18:1), suggesting shifts in mammary FA synthesis that support higher milk fat yields (Benoit et al., 2026) and help maintain milk fat fluidity.

High-oleic soybeans (containing ∼70% oleic acid [*cis*-9 C18:1]) are a promising on-farm feed, and a relatively new ingredient in dairy diets, providing an unsaturated fat source with lower risk of milk fat depression than conventional soybeans, which are high in linoleic acid. They also provide dairy producers with an affordable source of fat and protein. Recent studies showed that including roasted high-oleic soybeans at ∼16% of diet DM increased milk and milk component yields (Bales and Lock, 2024a,b). Similarly, supplying 18-carbon FA mainly as *cis*-9 C18:1 from high-oleic soybeans, while balancing 16-carbon FA intake, increased milk and component yields and improved feed efficiency compared with supplying C18:0 from a C18:0-enriched supplement (Seelenbinder et al., 2026). Roasting high-oleic soybeans enhances their nutritive value and improved milk production responses compared with feeding them raw (Bales and Lock, 2024a), in part by increasing RUP and reducing antinutritional factors (Grummer et al., 1994; Real-Guerra et al., 2013). Although current research is limited, high-oleic soybeans either do not affect or slightly decrease de novo milk FA yield while increasing C4:0, *cis*-9 C18:1, and preformed FA yields (Bales and Lock, 2024a,b), indicating their potential to support milk fat synthesis.

Although fat supplements blending C16:0 and *cis*-9 C18:1 have shown positive effects on milk production (de Souza et al., 2019, 2021; Lock et al., 2025), no studies have specifically evaluated feeding a C16:0 supplement together with high-oleic soybeans in the same diet. Benoit et al. (2024), for example, combined a C16:0 supplement with whole cottonseed (high linoleic and low oleic), but the use of high-oleic soybeans provides a different, oleic-rich profile. Therefore, we hypothesized that supplementing mid-lactation diets with (1) a C16:0-enriched FA supplement and (2) high-oleic soybeans would each increase milk and component yields and shift milk FA composition, and that feeding them together would further enhance these responses beyond either alone.

To test this hypothesis, we conducted a 4 × 4 Latin square experiment (14-d periods) with 20 multiparous mid-lactation Holstein cows (136 ± 18 DIM; 45.9 ± 4.7 kg/d milk) at the Michigan State University Dairy Cattle and Teaching Research Farm (East Lansing, MI) in January through March of 2024. Cows were housed in tiestalls with free access to water and milked thrice daily. After a 7-d preliminary period on a common diet, cows were blocked by milk yield and randomly assigned to one of 4 diets in a 2 × 2 factorial arrangement of a C16:0 acid supplement (**PA**) and high-oleic soybeans (**HOS**). The 4 treatments were (1) control (**CON**; no PA or HOS), (2) PA (1.5% of diet DM as a C16:0-enriched supplement), (3) HOS (16% of diet DM as roasted, ground high-oleic soybeans), and (4) PA + HOS (1.5% C16:0 + 16% HOS). The C16:0-enriched fat supplement replaced soybean hulls, and the high-oleic soybeans replaced conventional soybean meal, soybean-based bypass protein, and soybean hulls to maintain similar dietary nutrients of NDF, forage NDF, starch, CP, and RUP across treatments. Consequently, total dietary FA differed across treatments, with values being 1.56%, 2.92%, 4.03%, and 5.42% DM, for CON, PA, HOS, and PA + HOS, respectively (Table 1).

**Table 1:** Ingredient and nutrient composition of treatment diets^1^

Item	Treatment			
	CON	PA	HOS	PA + HOS
Ingredient, % DM				
Corn silage	41.6	41.6	41.6	41.6
Alfalfa silage	10.0	10.0	10.0	10.0
Alfalfa hay	1.86	1.86	1.85	1.86
Ground corn	12.5	12.5	12.5	12.5
Mineral and vitamin mix^2^	2.05	2.04	2.04	2.04
Lactation mix^3^	3.93	3.93	3.93	3.93
DCAD supplement^4^	0.47	0.47	0.47	0.47
Soybean meal	7.2	7.2	5.96	5.91
Soybean hulls	9.37	8.09	8.36	4.41
Soybean-based bypass protein^5^	11.1	11.0	—	—
High-oleic soybeans^6^	—	—	16.0	15.9
C16:0-enriched prill^7^	—	1.42	—	1.46
Nutrient composition, % DM				
NDF	31.5	30.6	29.4	28.3
Forage NDF	21.1	21.1	21.1	21.1
Starch	24.5	24.5	24.5	24.5
CP	17.1	16.9	17.3	16.7
FA	1.56	2.92	4.03	5.42
16:0	0.23	1.45	0.35	1.61
18:0	0.04	0.07	0.12	0.15
* cis*-9 18:1	0.28	0.36	2.46	2.52
* cis*-9, *cis*-12 18:2	0.85	0.86	0.89	0.90
* cis*-9, *cis*-12, *cis*-15 18:3	0.11	0.11	0.14	0.14

1 Nutrient composition values are based on composite samples collected during each experimental period and are presented as treatment means.

2 Contained 49.9% ground corn; 26.2% sodium sesquicarbonate; 12.1% calcium carbonate; 7.5% calcium phosphate; 2.5% urea; and 1.7% rumen-protected Met (Smartamine M, Adisseo, Alpharetta, GA).

3 Contained 20.8% calcium carbonate; 16.0% calcium magnesium carbonate (MIN-AD, Min-Ad Inc., Winnemucca, NV); 14.4% white salt; 13.3% ground corn; 12.7% calcium phosphate; 9.3% magnesium oxide; 4.8% magnesium sulfate; 4.5% sodium sesquicarbonate; 2.4% selenium; 0.7% tallow; and <1% ferrous sulfate, trace mineral mix (Micro 5; Selko, Indianapolis, IN), rumen-protected Met (Smartamine M, Adisseo, Alpharetta, GA), vitamin D, vitamin E, and vitamin A.

4 Ion Plus (D&D Ingredients LLC, Delphos, OH) contained 48.5% potassium and 0.31% sodium.

5 AminoPlus (Ag Processing Inc., Omaha, NE).

6 Plenish soybeans (Pioneer, Johnston, IA). FA profile (16.6% DM of total FA) consisted of (g/100 g FA): 5.09 C16:0, 3.05 C18:0, 83.0 *cis*-9 C18:1, 5.38 *cis*-9, *cis*-12 C18:2, and 1.53 *cis*-9, *cis*-12, *cis*-15 C18:3.

7 Propalm 85 (Perdue AgriBusiness, Salisbury, MD). The supplement (96.6% total FA content % DM) contained (g/100 g FA): 1.24 C14:0, 89.1 C16:0, 1.96 C18:0, 5.85 *cis*-9 C18:1, and 1.13 *cis*-9, *cis*-12 C18:2.

The high-oleic soybeans were processed with a flame roaster and hammer mill. Soybeans were heated to temperatures between 143°C and 149°C, steeped for 30 min (Hsu and Satter, 1995), and analyzed for grind size (748 ± 2.57 μm; Cumberland Valley Analytical Services, Waynesboro, PA). Additional samples were not collected to assess roasting quality, including protein dispersion index or RUP; however, the soybeans were processed using the same procedure as in Seelenbinder et al. (2026).

During the final 4 d of each period, feed intake was recorded and samples of dietary ingredients were analyzed for NDF, starch, CP, and FA content according to standard methods (Boerman et al., 2017; Bales et al., 2024). Milk yield was recorded at each milking, and milk samples were analyzed for fat, true protein, and lactose by mid-infrared spectroscopy (CentralStar Michigan Lab, Grand Ledge, MI). Milk lipids were extracted and FA methyl esters prepared for gas chromatography analysis (Bales and Lock, 2024b). Yields of individual milk FA were calculated from milk output and composition data (Benoit et al., 2024). Body weight was recorded directly following the afternoon milking on the last 2 d of each period, and BCS was measured on the last day of each period by 3 trained investigators on a 5-point scale (Wildman et al., 1982).

Data were analyzed using the PROC GLIMMIX model procedure of SAS (version 9.4; SAS Institute, Cary, NC). The statistical model included the random effect of cow within square, the fixed effects of period, square, PA, and HOS, and the interaction of PA × HOS. Interactions between PA and period, HOS and period, and PA × HOS and period were evaluated initially. Because none were significant (all *P* > 0.20), these terms were excluded from the final model. Normality was assessed using box plots and normal probability plots, and homogeneity of variances was verified. Treatment effects were declared significant at *P* ≤ 0.05 and tendencies at 0.05 < *P* ≤ 0.10. Results are presented as LSM ± SEM.

Because PA × HOS interactions were not significant for major production outcomes, including DMI and yields of milk and milk components (*P* > 0.10), responses were interpreted primarily according to the main effects of PA and HOS, although least squares means for all 4 treatment combinations are presented in Table 2. The HOS diet increased DMI by ∼1 kg/d *(P* < 0.05) and increased milk yield by ∼2.7 kg/d on average, relative to diets without HOS (*P* < 0.001). In contrast, PA did not affect DMI (*P* > 0.90) and tended to increase milk yield by ∼0.7 kg/d (*P* = 0.07). Milk fat yield responded strongly to both factors: PA increased milk fat yield by ∼90 g/d (+8% vs. control; *P* < 0.01), and HOS increased it by ∼170 g/d (+15%; *P* < 0.001). Cows fed PA + HOS produced the greatest milk fat yield, approximately +310 g/d relative to control (Table 2), which exceeded either treatment alone, demonstrating an additive benefit to milk fat yield. Milk fat percentage did not differ among treatments (*P* > 0.10) because increases in fat yield paralleled higher milk volume. The HOS diet increased milk protein yield by ∼0.2 kg/d *(P* < 0.01), whereas PA had no effect on protein yield (*P* > 0.20); consequently, HOS slightly reduced milk protein percentage due to dilution (*P* < 0.05), despite greater protein yield.

**Table 2:** DMI and production responses of cows fed treatment diets (n = 20)^1^

Variable	Treatment^2^				SEM^4^	*P*-value^3^		
	CON	PA	HOS	PA + HOS		PA	HOS	PA × HOS
DMI, kg/d	31.6	31.5	32.1	32.3	0.46	0.99	0.02	0.58
Yield, kg/d								
Milk	41.2	42.3	44.2	44.5	0.59	0.07	<0.001	0.29
3.5% FCM^5^	44.3	46.3	49.3	50.7	0.78	<0.001	<0.001	0.68
ECM^6^	44.6	46.2	48.2	50.1	0.70	<0.001	<0.001	0.70
Fat	1.63	1.72	1.80	1.94	0.04	<0.001	<0.001	0.16
Protein^7^	1.41	1.44	1.46	1.46	0.02	0.21	<0.01	0.26
Lactose	2.01	2.05	2.20	2.20	0.03	0.34	<0.001	0.35
Milk composition, %								
Fat	4.00	4.16	4.10	4.41	0.08	<0.001	<0.001	0.13
Protein	3.45	3.38	3.32	3.32	0.03	0.02	<0.001	0.03
Lactose	4.89	4.90	4.96	4.95	0.02	0.91	<0.001	0.14
ECM/DMI	1.41	1.45	1.50	1.55	0.03	<0.001	<0.001	0.66
BW, kg	765	760	760	758	12.8	0.14	0.06	0.49
BW change, kg	13.4	4.07	4.76	1.54	2.8	0.03	0.05	0.27
BCS	3.05	3.07	3.11	3.16	0.05	0.14	0.01	0.57
BCS change	0.01	0.03	0.05	0.05	0.04	0.77	0.43	0.76

1 Experimental diets fed to 20 multiparous cows in a 2 × 2 factorial arrangement in a 4 × 4 Latin square design with 14-d periods that were balanced for carryover effects. Samples and data for production variables collected during the last 4 d of each treatment period (d 10 to 14).

2 CON = no palmitic acid or high-oleic soybean supplementation; PA = 1.5% DM palmitic acid-enriched prill; HOS = 16% DM roasted and ground high-oleic soybean; PA + HOS = 1.5% palmitic acid-enriched prill + 16% DM roasted and ground high-oleic soybean.

3 Contrasts are as follows: PA = effect of treatment diets containing PA; HOS = effect of treatment diets containing HOS; PA × HOS = the interaction of PA and HOS.

4 Greatest SEM.

5 Fat-corrected milk: 3.5% FCM = (0.4324 × kg milk) + (16.216 × kg milk fat).

6 Energy-corrected milk: ECM = (0.327 × kg milk) + (12.95 × kg milk fat) + (7.20 × kg milk protein). This equation corrects milk to a 0.68-Mcal/kg energy basis.

7 Protein values represent milk true protein concentration and yield.

Milk FA yields by source differed according to the FA substrates supplied by the treatment diets (Figure 1). Individual milk FA yields are presented in Supplemental Table S1 (see Notes). Neither PA nor HOS altered de novo FA yield (<C16; ∼430 g/d; *P* > 0.20). The PA diet increased the yield of 16-carbon (mixed) FA in milk fat (*P* < 0.05), whereas HOS tended to reduce mixed FA yield, resulting in a PA × HOS interaction for this group (*P* < 0.05). The HOS diet increased preformed FA yield (>C16) from ∼470 to ∼740 g/d (*P* < 0.001), whereas PA had little effect on preformed FA (*P* = 0.77). Together, PA and HOS increased milk fat yield by increasing complementary FA pools: PA increased mixed FA yield, whereas HOS increased preformed FA yield, explaining the substantially greater total milk fat yield observed with the combined treatment.

**Figure 1:**
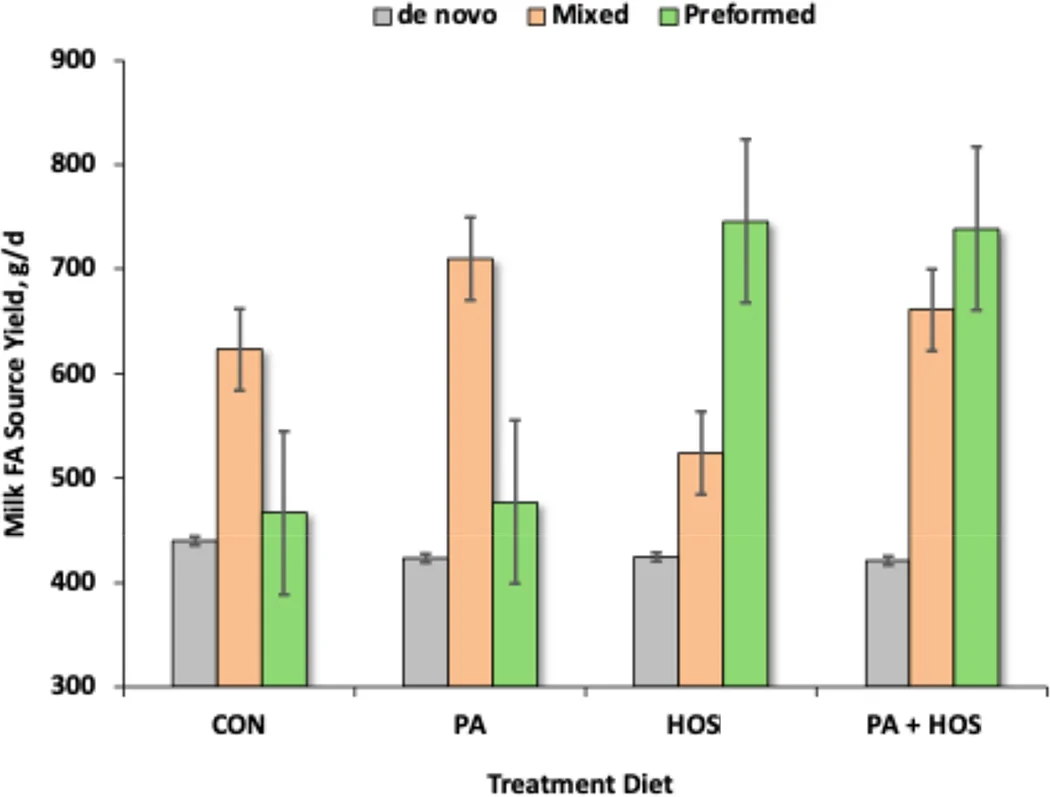
The effects of treatment diets on the yields of de novo, mixed, and preformed milk FA sources. Experimental diets fed to 20 multiparous cows in a 2 × 2 factorial arrangement in a 4 × 4 Latin square design with 14-d periods that were balanced for carryover effects. Samples and data for production variables collected during the last 4 d of each treatment period (d 10 to 14). Treatments were as follows: CON = no palmitic acid or high-oleic soybean supplementation; PA = 1.5% DM palmitic acid-enriched prill; HOS = 16% DM roasted and ground high-oleic soybean; PA + HOS = 1.5% palmitic acid-enriched prill + 16% DM roasted and ground high-oleic soybean. Significance was determined at *P* ≤ 0.05, and tendencies were determined at *P* ≤ 0.10. Contrasts are as follows: PA = effect of treatment diets containing PA; HOS = effect of treatment diets containing HOS; PA × HOS = the interaction of PA and HOS. There was no effect of treatment on de novo milk FA yield (all *P* > 0.19). The PA-fed cows had increased mixed milk FA yields (*P* ≤ 0.001) but did not affect preformed milk FA yields (*P* = 0.77). The HOS-fed cows had decreased mixed milk FA yields (*P* ≤ 0.001) but increased preformed milk FA yields (*P* ≤ 0.001). Values are LSM ± SEM.

Palmitic acid supplementation increased milk fat yield by ∼8%, consistent with extensive evidence that C16:0 enhances milk fat production in mid-lactation cows (Rico et al., 2014; de Souza and Lock, 2018; dos Santos Neto et al., 2021). This response was driven by increased yields of 16-carbon (mixed) FA, supporting the role of C16:0 as a key substrate for initiating mammary TG synthesis through preferential esterification early in glycerol backbone assembly (Moore and Christie, 1979; Benoit et al., 2024). This facilitates subsequent esterification of other FA onto TG, effectively “priming” milk fat assembly. Accordingly, PA-fed cows had greater 16-carbon FA output. Although some studies have reported slight reductions in de novo FA yield with PA supplementation when expressed on a mass basis (g/d), Benoit et al. (2026) showed that these responses are not necessarily evident when de novo FA output is evaluated on a molar basis (mmol/d). In our mid-lactation setting, de novo FA yield remained unchanged, indicating that increased 16-carbon FA supply supported TG formation without requiring compensation through reduced short-chain FA synthesis.

High-oleic soybeans increased milk and milk component yields, including an approximately 15% increase in milk fat yield, consistent with previous studies in high-producing cows (Bales and Lock, 2024a,b). In the present study, HOS substantially increased preformed (>16-carbon) FA yield by increasing the supply of long-chain FA to the mammary gland. Previous high-oleic soybean studies similarly reported greater preformed milk FA yield with little or no reduction in de novo FA yield compared with non-FA-supplemented (Bales and Lock, 2024b) or FA-supplemented control diets (Seelenbinder et al., 2026). Because dietary *cis*-9 C18:1 is extensively biohydrogenated in the rumen to C18:0, HOS would have supplied both *cis*-9 C18:1 and C18:0 to the mammary gland, expanding the pool of preformed FA available for direct incorporation into milk fat and contributing to the ∼50% increase in preformed FA yield. Importantly, despite increasing total dietary fat to approximately 4% to 5%, HOS did not depress de novo FA synthesis, likely because moderate *cis*-9 C18:1 supplementation avoided the ruminal shifts associated with high-PUFA feeding, such as accumulation of *trans*-10 intermediates or reduced fiber digestion (Dorea and Armentano, 2017). These results indicate that high-oleic soybeans can provide energy-dense UFA and support milk fat production without compromising rumen function or short-chain FA synthesis.

The central finding of this study is that feeding PA and HOS together produced a greater milk fat response than either strategy alone. Cows fed the combined treatment produced ∼0.3 kg/d more milk fat than CON (about double the gain from PA or HOS alone), indicating that each FA source alleviated different constraints on milk fat synthesis. This result supports the concept that balanced supplies of de novo and preformed FA substrates maximize milk fat synthesis (Glasser et al., 2008; Benoit et al., 2024). By providing an abundance of C16:0 (whether preformed in the diet or via de novo pathways) along with additional long-chain FA (*cis*-9 C18:1 and C18:0), the mammary gland can incorporate both sources concurrently into TG. The supply of complementary substrates likely minimized any need for one FA source to substitute for the other, thus averting the typical trade-off where adding UFA often suppresses de novo FA output (Dorea and Armentano, 2017). These results support an interdependent model of milk fat synthesis in which short- and long-chain FA jointly drive TG assembly (Glasser et al., 2008; Gresti et al., 1993) and thereby total fat yield.

Our results closely parallel those observed when feeding mid-lactation cows, specifically high-producing cows (>45 kg/d of milk), with C16:0 and *cis*-9 C18:1 supplied as supplemental FA blends (de Souza et al., 2019; Western et al., 2020; Burch et al., 2021). Notably, no prior study has evaluated a C16:0 supplement plus a high-oleic oilseed in one experiment. Benoit et al. (2024) included C16:0 with whole cottonseed (rich in C18:2) and found that preformed C18:2 partly replaced de novo FA in milk fat, whereas our high-oleic soybean treatments did not depress de novo synthesis. The PA + HOS combination effectively delivered multiple complementary FA substrates to the mammary gland—C16:0 to stimulate TG formation and 18-carbon FA (*cis*-9 C18:1 and C18:0) to ensure ample long-chain FA incorporation—thereby overcoming any single-FA limitations. The outcome was a robust increase in milk fat yield beyond either strategy alone, highlighting the nutritional synergy of feeding these FA together when properly balanced.

The stable de novo FA yield across treatments suggests that mammary short-chain FA synthesis was not limiting, likely because rumen fermentation was adequate and dietary fat inclusion was moderate. Although high supplies of UFA can suppress de novo synthesis in other contexts (Glasser et al., 2008; Dorea and Armentano, 2017), the FA profile of high-oleic soybeans appeared to avoid this response in the present study. However, individual de novo FA shifted despite no change in total de novo FA yield, most notably through an additive increase in C4:0 when PA and HOS were fed together (Supplemental Table S1). Together with broader changes in short- and medium-chain milk FA, this pattern suggests that mammary FA synthesis was preserved but adjusted to support TG assembly and maintain milk fat fluidity at body temperature (Jensen et al., 1991). Thus, the marked increase in preformed (>16-carbon) FA uptake was likely accommodated by sufficient C16:0 and short-chain FA availability for co-esterification on the glycerol backbone, allowing each TG to contain the necessary mix of FA chain lengths. Overall, these responses support a coordinated-substrate model in which PA and HOS enhanced milk fat synthesis by increasing long-chain FA availability while preserving shorter-chain FA availability needed for efficient TG assembly (Benoit et al., 2024).

From an applied perspective, these results support the use of high-oleic soybeans alongside C16:0-enriched supplements in dairy rations to increase milk fat yields. Feeding 1.5% DM C16:0 + 16% DM high-oleic soybeans yielded ∼0.3 kg/d more milk fat than a non-FA-supplemented control, without negatively affecting DMI, milk protein yield, or body condition. This approach leverages a farm-grown oilseed alongside targeted C16:0 supplementation, improving TG synthesis and overall production efficiency. This study evaluated mid-lactation cows over relatively short experimental periods, which may not capture longer-term adaptation or responses across lactation stages. In addition, the focus on production and milk FA outcomes precluded direct assessment of rumen fermentation and nutrient digestibility. Future research should determine whether these additive responses persist during extended feeding and in early-lactation cows, where intake capacity and energy balance differ. Further work should also evaluate optimal inclusion rates and mechanistic responses related to FA uptake and TG synthesis. Addressing these questions will refine feeding recommendations and help establish the broader applicability of combining C16:0 with *cis*-9 C18:1 to enhance milk fat production across lactation stages and feeding systems.

In conclusion, feeding mid-lactation cows a combination of supplemental C16:0 and high-oleic soybeans increased milk fat yield more than either strategy alone. Palmitic acid supplied extra 16-carbon FA and high-oleic soybeans provided additional preformed FA, increasing milk fat output without suppressing milk de novo FA production. This balanced FA supply supported efficient mammary TG synthesis and drove an additive milk fat response. These findings demonstrate that combining a C16:0 supplement with high-oleic soybeans is an effective practical strategy to increase milk fat yield in mid-lactation dairy cows.
